# The use of the Video Platform FlipGrid for Practicing Science Oral Communication

**DOI:** 10.1007/s11528-022-00801-1

**Published:** 2022-10-25

**Authors:** Alexandros Kleftodimos, Amalia Triantafillidou

**Affiliations:** Department of Communication and Digital Media, University of Western Macedonia, 52100 Fourka Area Kastoria, Greece

**Keywords:** Flipgrid, Oral communication, Science Communication, Video Mediated Communication, Video Discussion Platforms, Video Based Learning

## Abstract

Oral communication and presentation skills are considered key competencies for many academic fields and professions, including science-related ones. Therefore, it is vital that students are given sufficient class time in the curriculum for practicing public speaking. It is well documented in the literature that video technology can be of valuable aid in enhancing oral skills, and FlipGrid is a relatively new video discussion platform that has become popular for this purpose. The aim of this study is two-fold. First, the literature regarding FlipGrid is explored to understand how educators around the globe use the platform. Second, a case study is presented where FlipGrid is used and evaluated as a tool for practicing and assessing oral science communication. A mixed-method approach was used to evaluate FlipGrid with the participation of 112 students. Findings indicate that although the use FlipGrid in science fields is limited, it can be a useful platform for practicing oral science communication.

## Introduction

Oral communication and presentation skills are recognized as key competencies across many academic and professional fields and are also essential for science communication.

Scientists with effective communication skills can contribute to making science more accessible to the public and help in improving people’s scientific literacy (Oliveira et al., 2021). Scientific findings should also be communicated effectively to politicians and policymakers to aid their decision-making process (Brownell et al., 2013; Chan, 2011; Oliveira et al., 2021). In order to take advantage of funding opportunities, scientists must be able to communicate to reviewers and the scientific community how their research is valuable and applicable to society (Feliú-Mójer, 2015; McNutt, 2013). Furthermore, scientists must also be capable of communicating complex science concepts to non-scientific audiences by using simple and understandable language since policy and funding decisions can also depend on public understanding of science (Brownell et al., 2013; Ponzio et al., 2018). There is a large number of studies published over the years that pinpoint the necessity for science graduates to be effective communicators (e.g., Brownell et al., 2013; Burns et al., 2003; Chan, 2011; Cormier & Langlois, 2022; Feliú-Mójer, 2015; Haworth & Garrill, 2003; Leshner, 2007; McDonald & McDonald, 1993; McNutt, 2013; Mercer-Mapstone & Kuchel, 2017; Neeley et al., 2015; Oliveira et al., 2021; Ponzio et al., 2018; Purnomo & Fauziah, 2018; Reitmeier et al., 2004).

There are many definitions encountered in the literature regarding science communication. Oliveira et al., (2021) claim that science communication can be loosely defined as any activity that involves one person orally sharing science-related information with another. But there are also more detailed definitions. According to Burns et al. (2003) science communication (SciCom) is defined as the use of appropriate skills, media activities, and dialogue to produce one or more of the following personal responses to science: awareness, enjoyment, interest, opinion-forming, and understanding. Mercer-Mapstone and Kuchel, (2017), on the other hand, define science communication as the process of translating complex science into language and concepts that are engaging and understandable to non-scientific audiences such as politicians, industry professionals, journalists, government, educators, business, and the lay public.

Communication is a skillset that is highlighted consistently by educators, employers, government, and professionals as being highly important for science graduates (Brownell et al., 2013; Mercer-Mapstone & Kuchel, 2017; West, 2012). Communication has been recommended as an important learning outcome for science degrees in countries such as Australia, the UK, the US, and Canada (Mercer-Mapstone & Kuchel, 2017).

There are two main ways of communicating scientific information: orally and in writing. Effective oral communication, which is the focus of this study, is important for the progress and dissemination of scientific knowledge. There is increasing recognition within higher education institutions of the need for science graduates to be equipped with solid oral and presentation skills (Chan, 2011). Effective oral communication is not an innate ability but an ability that must be developed through practice (Cormier & Langlois, 2022). Anxiety and fear of public speaking can also be overcome by practice. Thus, it is necessary to develop and incorporate into the learning process constructive learning tasks that would enable and motivate students to develop their oral communication and presentation skills (Chan, 2011). Oral skills can be cultivated in class and through activities such as PowerPoint and poster presentations, as well as student-driven tutorials and discussions (Chan, 2011). However, the major disadvantages of such activities are the logistics and time and workload implications involved, especially for large undergraduate classes (Chan, 2011). Two possible ways to overcome these difficulties are splitting large audiences into smaller groups and employing more trainers or having students submit their oral presentations electronically in video form (Chan, 2011).

Much debate also exists over how to incorporate communication skills teaching and practice in science degrees. One approach is introducing new dedicated courses in the curriculum, and another is incorporating the teaching and practice of communication skills into existing courses. Many educators are in favor of the latter. Integrating communication skills into existing courses can mean communication is taught in a discipline-specific context (Mercer-Mapstone & Kuchel, 2017).

Besides the need for practicing oral and written communication, there are also other skills encountered in the literature that are useful for science communication. Mercer-Mapstone and Kuchel (2017) have produced a list of core skills by examining the literature and using expert help to validate the results. Amongst these skills is the ability to understand the target audience and use appropriate language for that audience, to use storytelling techniques effectively, to use style elements such as humor, anecdotes, and imagery, and to employ a suitable platform for dissemination, etc.

Although it is broadly accepted that oral communication across many scientific fields is an important skill, it is often under-taught and under-assessed. On the contrary, written communication is often assessed through the curriculum with methods such as written assignments and written exams (Chan, 2011; Haworth & Garrill, 2003; Race, 2007). Reserving time for developing these competencies in the curriculum, however, is not easy and depends on the educational setting. As already mentioned, it isn’t easy to practice oral communication in large classes. In most cases, only a few students can express their thoughts orally or practice their verbal presentation skills in front of an audience due to time constraints and the pressure that the educator has to cover the syllabus topics. This problem is also present in distant education settings where synchronous video conferencing platforms are used (e.g., Zoom and Webex).

Video recording software can be used to cope with the problem of insufficient time allocation for oral communication and presentation practice. After a presentation is recorded and stored in the system, students can reflect on their performance and receive feedback from their lecturers and classmates if this is desirable. Video reflection on communicative performance has been used successfully in many academic and professional fields, such as language learning, medical studies teacher training, engineering, and economics, as well as other science and social studies fields (e.g., Budiarta & Santosa, 2020; Carr et al., 2020; Cochrane & O’Donoghue, 2008; Er & Planas, 2005; Galindo et al., 2020; Gong et al., 2019; Miskam & Saidalvi, 2020; Oliveira, et al., 2021; Penny & Coe, 2004; Perry et al., 2020; Ram, et al., 1999; Zick et al., 2007). By reflecting on a video presentation, students can pinpoint their strengths and weaknesses in all aspects of the presentation, including the content (Miskam & Saidalvi, 2019, 2020; Oliveira, et al., 2021; Tuyet & Khang, 2020). Moreover, with video recordings, verbal communication can be practiced in a controlled, non-threatening environment that helps alleviate anxiety (Tuyet & Khang, 2020; Damayanti & Citraningrum, 2021; Göktürk, 2016).

Of course, challenges are also reported in the literature regarding video technology as a tool for oral presentations. Although video-mediated environments used for self-reflection can help reduce anxiety, the opposite is also reported in some studies. Anxiety triggered when speaking in front of the camera appeared to be an issue for some students (Keiper et al., 2021; Miskam & Saidalvi, 2020; Oliveira et al., 2021). Other studies found that students faced technical difficulties recording videos (Göktürk, 2016; Miskam & Saidalvi, 2020; Shih, 2010; Syahrizal & Pamungkas, 2021).

Various solutions are encountered in the literature regarding the software used for recording and hosting video presentations and discussions. These solutions range from simple software programs for recording and storing videos on a hard drive or an optical disk (CD & DVD ROM), to video-based blogs (V-logs) (Sukiman, 2016) and other more advanced video-mediated communication (VMC) tools such as Virtual-i Presenter (Cochrane & O’Donoghue, 2008), Vialogues (Agarwala et al., 2012; Zafar, 2020), and Voicethread (Delmas, 2017).

Flipgrid is a relatively new platform that has become popular in recent years in the educational community. Researchers at the University of Minnesota created FlipGrid, and in June 2018, Microsoft acquired FlipGrid and made the platform freely available to educators worldwide (Coss, 2018). FlipGrid is used primarily as a tool for hosting video discussions and peer-to-peer communication but can also serve as a tool for oral communication tasks assigned by educators.

This study aims to explore the efficiency of FlipGrid as a tool for practicing scientific oral communication. First, the relevant literature will be investigated to map the academic disciplines where FlipGrid is used. The literature review will also examine how the platform is used by educators and researchers worldwide and the extent to which FlipGrid is used for practicing science oral communication. Then a case study will be presented where FlipGrid is used and evaluated in an educational setting as a tool for oral assignments in a science-related course.

More specifically, in Section 2, a review of the scientific literature regarding this specific platform is conducted. Section 3 presents a case study where FlipGrid is used and evaluated in an educational setting as a tool for practicing and assessing scientific oral communication. The educational setting, the data collection methods, and the hypotheses to be tested are explained in this section. Section 4 presents the results obtained from the statistical analysis of the data as well as observations and results from a content analysis of student comments. A summary of the findings and the limitations of the study are presented in Section 5. The paper concludes in Section 6.

## Exploring the Scientific Literature for Studies that use the FlipGrid Platform

FlipGrid, as mentioned in the introduction, is a video discussion platform that has become popular amongst educators. The reason for this is some of the unique capabilities this platform offers for facilitating oral & presentation activities and discussions. FlipGrid can be accessed through its website (flipgrid.com), but a FlipGrid mobile version is also available for download from Microsoft, the App Store (Apple), and Google Play. Accounts can also be created by using Google or Microsoft credentials. FlipGrid can also be integrated within platforms such as Google Classroom and Microsoft Teams.

As far as the students are concerned, the platform’s user interface is intuitive and user-friendly and functions similarly to other video-based social media platforms that the young generation is familiar with (e.g., TikTok, Snapchat, etc.). More specifically, a social-media-style camera with editing options is used to record and upload videos. The Camera tool also provides the user with additional options, such as effects that can be overlayed to the video (e.g., filters, text, pen drawings, images, etc.) as well as background effects (e.g., blur, images). Editing capabilities are also present. Furthermore, the platform allows educators and students to carry out screen recordings (either full-screen or split-screen). This feature has opened new opportunities for presentation assignments since PowerPoint files can be used in video recordings.

The platform also provides the educator with an administrator environment (called “Educator Dashboard”) that incorporates many useful management capabilities. Using FlipGrid, educators can create Groups and invite students to join a Group. FlipGrid provides the educator with many invitation options (e.g., invitation by email, QRcodes, invitations through Google classroom, etc.). Educators can then create topics within a Group. Topics are conversation prompts with a question or idea, experiment, debate, oral assignment, or anything the educator can think of to ignite a conversation.

Educators can take batch actions on all student video responses in a topic but also actions on specific responses. For example, the educator can decide: whether a topic is open for new submissions, whether student video responses are visible to other students or just the educator, whether video responses are open for “likes”, written comments, or video comments, whether video responses can be downloaded, whether students can embed effects to their videos and so on.

There is also a lot of support for educators that wish to use FlipGrid. The FlipGrid website offers many resources that can aid students and educators in operating the software (e.g., a detailed guide by Fahey et al., 2019, webinars, etc.), as well as many guidelines and tips for integrating the software into the educational process. Furthermore, there is an informative blog (blog.flipgrid.com) and an active community on Twitter where educators share ideas, experiences, pedagogical tips, and strategies. A quick search using the hashtag “#FlipGrid” on Twitter returns an abundance of results from educators around the world who are using the platform in their classes.

All of the above affordances have made FlipGrid a popular video discussion platform for educational purposes.

According to the literature, FlipGrid has been used in many domains and for various purposes. An effort will be made to answer the following research questions by examining the related literature: a) is research about the use of FlipGrid in education increasing since 2018, when Microsoft acquired FlipGrid? b) in what educational fields is FlipGrid used, and for which purposes? c) is FlipGrid used for developing oral communication and presentation skills in science-related fields? e) what are the countries of these research studies, and which levels of education do these studies concern? f) what are the research methods used in these studies? g) what advantages and disadvantages are reported in the literature regarding the use of the platform?

### Searching Methodology and Exclusion Criteria

First, we searched the Scholar Google database using the keyword “FlipGrid”. We believe that since we are looking for papers concerning the use of this platform, the specific search term will suffice to return the expected results. We also searched the Scopus, Springer, Eric, ENSCO, and IEEEXplore database to check if other papers were missed from the Scholar Google search. The search aimed to spot scientific articles published in journals, conferences, and book chapters that contain the word “FlipGrid” in one of the following places: the title and the abstract of the manuscript, and the text snippet of the search results.

The search took place in October 2021 and yielded 46 articles. From these, we filtered out media articles, technical reports, and dissertations. After reading the abstract and the methodology section of these articles, more articles were excluded since some did not conduct research using the tool. After the filtering, we ended up with 29 articles. Almost all the papers (apart from one) contained the word FlipGrid in the title or the abstract. The articles published in scientific journals were 25, and four articles were published in Conference proceedings. All these papers are presented in Appendix A, Table 3. Table 3 also contains the research’s scientific field, the study’s aim, the research methods and data collection instruments, the educational level (K12, Higher Education), the country where the study took place, and the number of participants in the research.

### Results and Discussion

**Fig. 1:**
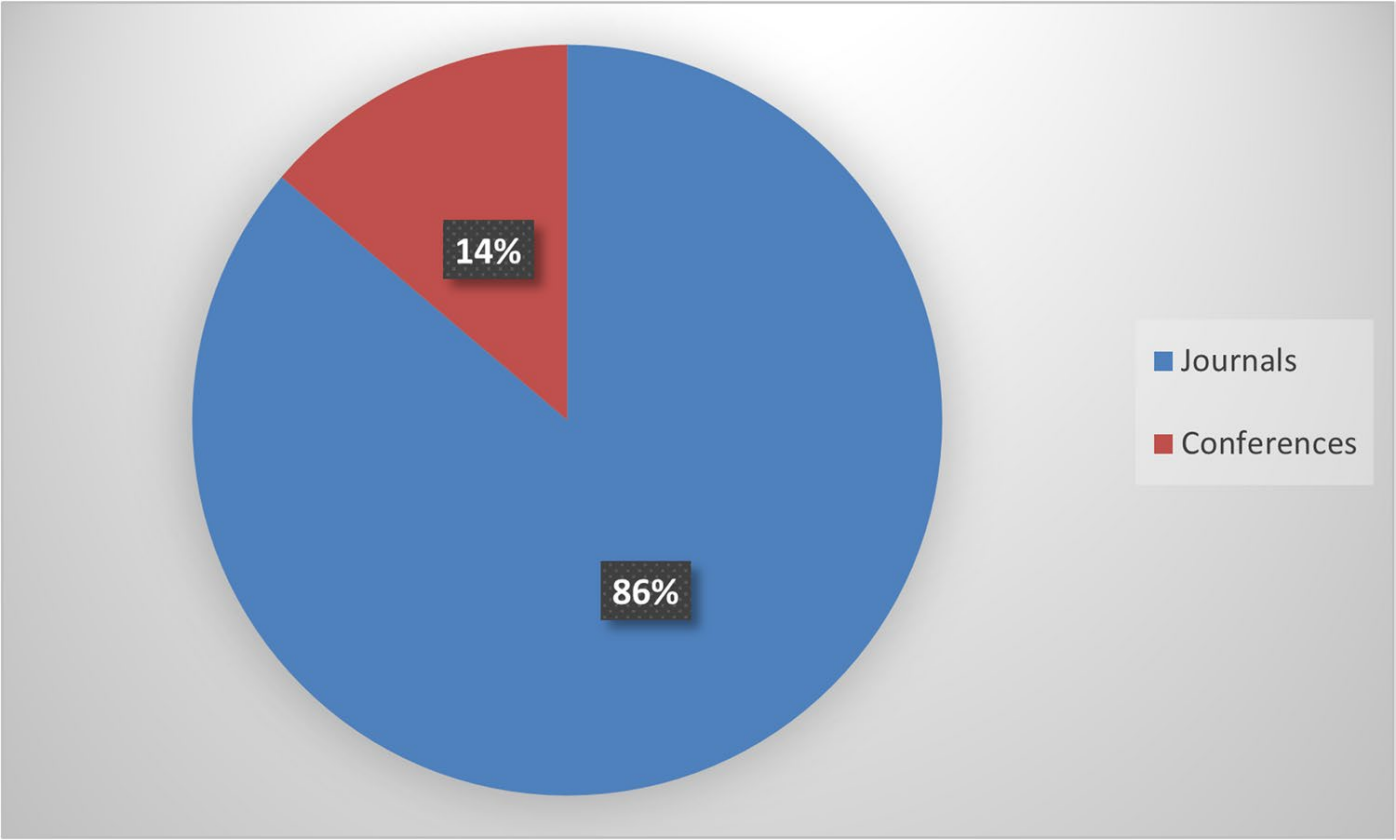
Year of Publication

As mentioned in the previous section, 25 articles were published in scientific journals, and four in Conference proceedings (Fig. 1).

**Fig. 2:**
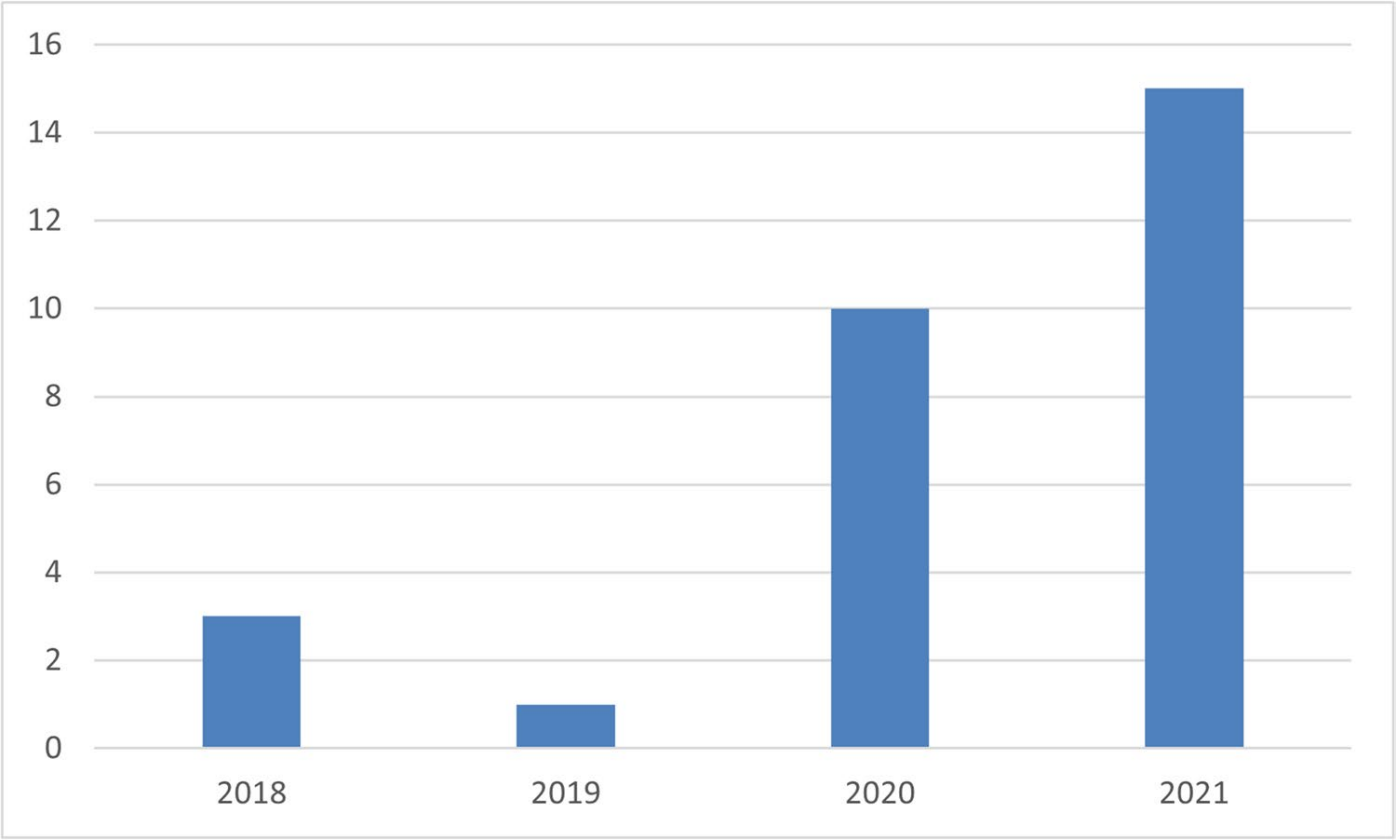
Year

Concerning the year of publication, these articles are all published after 2018 since FlipGrid is a relatively new platform. As mentioned, it became known to the educational world after Microsoft acquired it in 2018. It is evident from the graph in Fig. 2 that most of the research conducted using FlipGrid is very recent, and most articles were published in 2020 and 2021. It is reasonable to assume that COVID-19 and the need for innovative asynchronous video tools played an essential role in this. It is worth mentioning that 29 articles in this limited time frame is a large number also considering that these articles concern a specific software platform. It is thus evident that FlipGrid has caught the attention of educators.

**Fig. 3:**
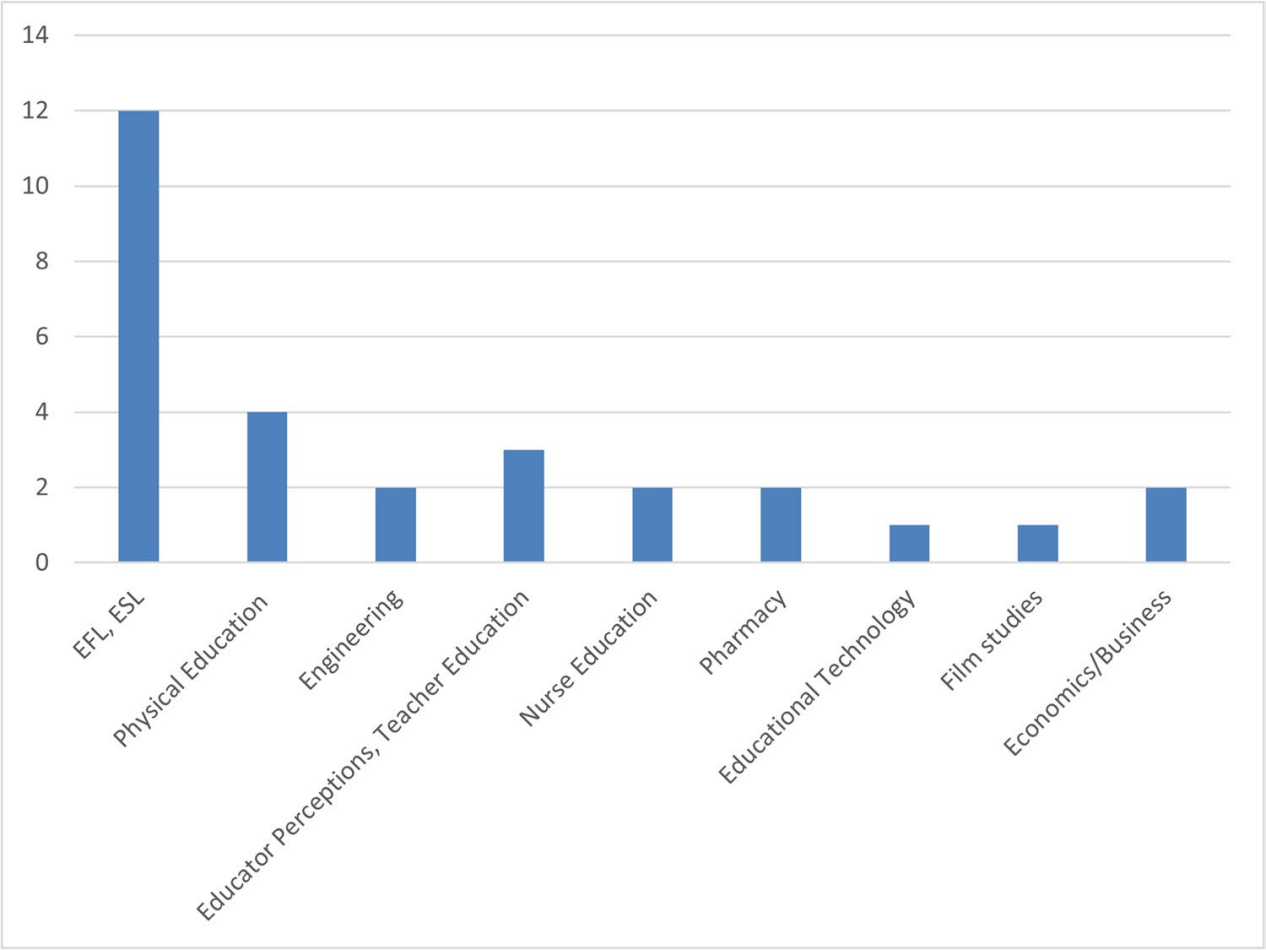
Field of Study

As far as the disciplines of the studies are concerned, it is obvious from the graph in Fig. 3 that most of the studies (12 articles) are in the field of language learning (EFL/ESL) (e.g., Iglesias, 2021; Mango, 2021; Petersen et al., 2020). FlipGrid proved to be particularly popular in foreign language learning and for practicing oral skills.

FlipGrid is also used in Physical Education (four papers) (Stoszkowski, 2018; Stoszkowski & Collins, 2021; Stoszkowski et al., 2021; Taylor & Hinchman, 2020). There is a need in Physical Education studies for students to demonstrate motor skills competency. Students may learn better the necessary motor skills by teaching these skills to their peers and by observing their peers while they demonstrate these skills (Taylor & Hinchman, 2020). Using Flipgrid, students can practice performing and teaching a skill while being recorded, granting the instructor and peers an opportunity to review and offer suggestions for improvement. Students can also self-reflect on their recordings to improve their motor skills.

As can be seen in Fig. 3, FlipGrid is also used to a lesser extent in other scientific fields such as Engineering (Ismail & Omar, 2020; Miskam & Saidalvi, 2019), Nurse education (Davis & Colella, 2021; Serembus & Murphy, 2020), and Economics and Business studies (Carr & Kruggel, 2020; Keiper et al., 2021). It is evident from the above graph that the use of FlipGrid is almost nonexistent in many traditional scientific fields such as Maths, Informatics, Physics, Chemistry, etc. Another striking observation is that FlipGrid is not utilized in Communication studies where public speaking skills are essential to the profession. Journalism studies, for example, could benefit from integrating a tool like FlipGrid into the educational process.

**Fig. 4:**
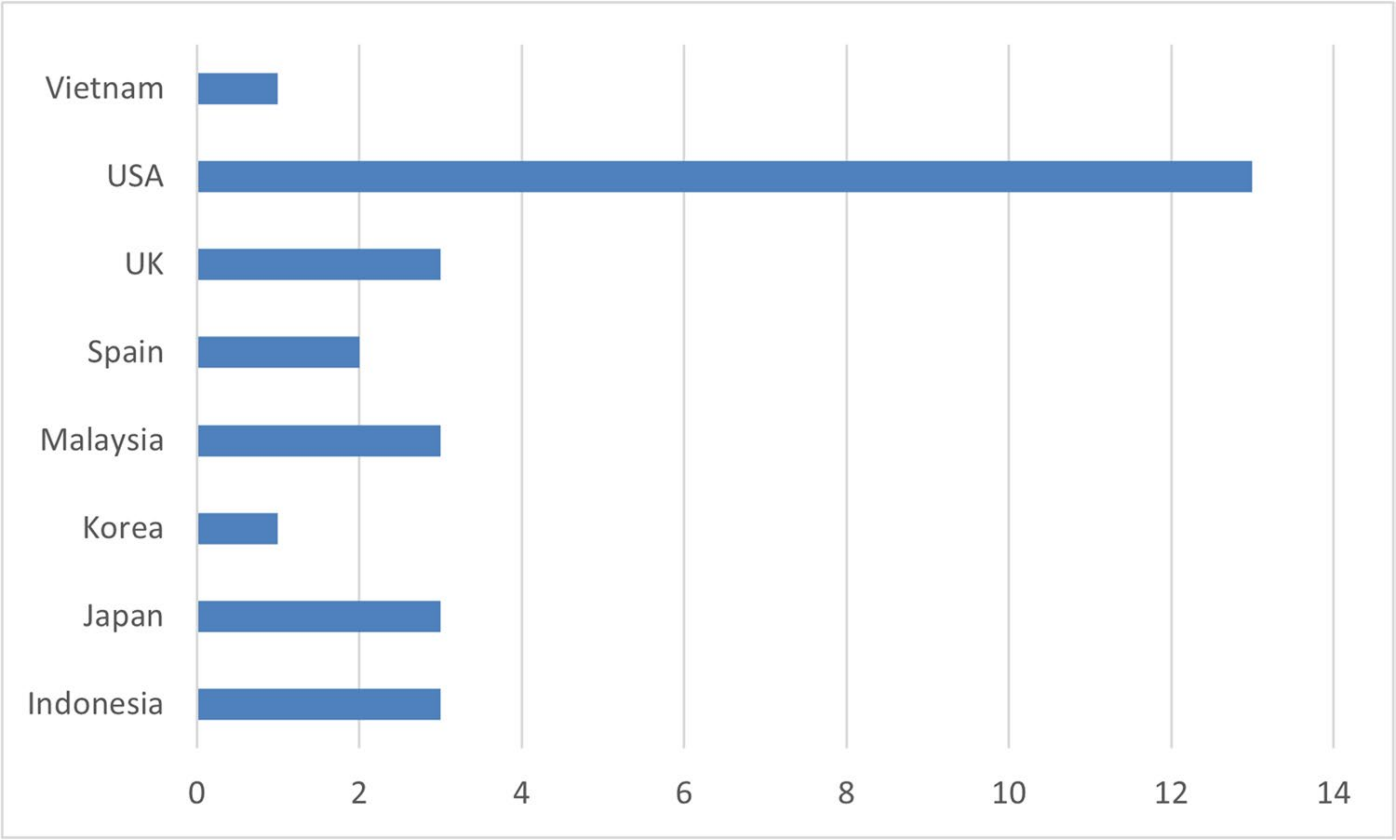
Countries of Research Papers

The studies were carried out in various countries, and a large percentage of the pie belongs to the USA (47%), as is depicted in Fig. 4.

**Fig. 5:**
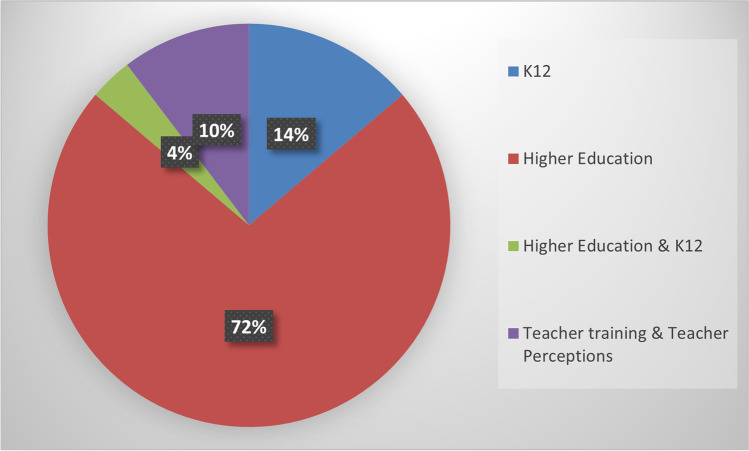
Educational Level

Most studies are conducted in higher education settings (21 studies), and only a few concern K12 education (four studies). There is also one study that concerns both higher and K12 education. Typically, in these studies, a sample of students is employed to research student attitudes and perceptions regarding the platform. Furthermore, three studies concern teacher training and teachers’ perceptions about FlipGrid (Fig. 5).

**Fig. 6:**
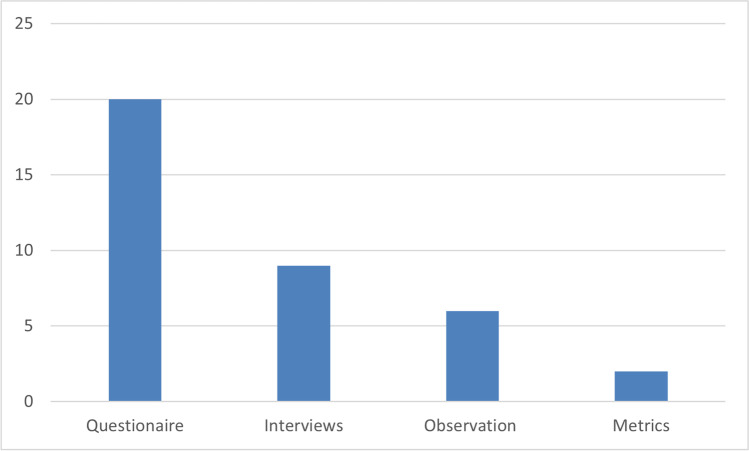
Data Collection Methods

The survey was the main method employed for recording student perceptions (20 studies). Various types of questionnaires were used to capture student experience, consisting mainly of Likert-scale and open-ended questions. Interviews were also utilized in nine studies mainly in combination with a survey. Three studies examined metrics such as the number of videos, length of videos, replies, number of views, etc., and three studies performed content analysis (e.g., on video transcripts and web resources). Some studies also relied on researcher observations, usually in combination with other methods such as surveys and interviews. Two studies expressed only personal opinions and experiences without using any particular research method (Fig. 6).

The survey sample sizes varied from small samples of around 20 students to a larger sample of 230 participants, as shown in Table 3. However, only six studies used a sample size of 100 and more students (Edwards & Lane, 2021; Green et al., 2021; Innes, 2020; Keiper et al., 2021; McLain, 2018; Syahrizal & Pamungkas, 2021). Thus, we can conclude that there is a lack of studies regarding FlipGrid that involve large sample sizes (e.g., ≥ 100).

By examining these papers, we conclude that FlipGrid is utilized mainly for two reasons. a) for improving oral skills (mainly in EFL/ESL) through self-reflection and feedback from the instructor or peer students and b) for initiating discussions that contribute to community building (e.g., Lowenthal & Moore, 2020; Serembus & Murphy, 2020). In the era of COVID-19, a tool such as FlipGrid proved helpful in breaking student isolation. As far as discussions are concerned, video communication poses advantages over written communication on discussion boards. Verbal, visual, and tonal cues, as well as facial expressions, make conversations less ambiguous and strengthen engagement among students (Wood, 2021). Tone and intention are typically harder to decipher or easily confused in written discussions. Engagement and connectedness became significant issues during the COVID-19 pandemic, a period in which many students felt isolated in their new online learning environments (Wood, 2021).

FlipGrid is also used for collaborative learning (e.g., Stoszkowski, 2018; Stoszkowski & Collins, 2021; Stoszkowski et al., 2021). Other purposes of FlipGrid use include “class introductions”, “debate activities”, as well as oral assignments (Ismail & Omar, 2020). In “class introductions,” the educator and the students record a short video introducing themselves, so everyone gets to know each other. In “debate activities”, the educator posts a debate topic, and students argue on different sides. Oral assignments and presentations through FlipGrid increase instructor awareness of student understanding of course concepts. FlipGrid also provides an effective method of formative assessment that prevents impersonation, which is a difficult problem to tackle in written assignments.

Considering all the above features, the advantages of FlipGrid as a tool for social learning, community building, and oral assignments become evident since it provides a learning space where students can easily record, edit, and upload their responses. Despite the advantages that Fliprid poses, there are also disadvantages and concerns reported in the literature. One possible disadvantage of FlipGrid as a tool for developing oral communication skills is that it allows students to edit their videos to produce a good presentation. Although this can be a desired feature for the students, it can also be viewed as a disadvantage, as Cochrane, and O’Donoghue (2008) mention in their study. The authors claim that video or audio editing capabilities do not help students improve their oral presentation skills. Other disadvantages include a feeling of competitiveness and stress especially when videos include the features of “likes” and “dislikes” (Syahrizal & Pamungkas, 2021). Anxiety and feeling uncomfortable about being “on screen” have also been reported by a few students (Keiper et al., 2021; Syahrizal & Pamungkas, 2021). Other problems reported concern technical difficulties associated with inadequate digital devices (e.g., camera, microphone, computer, or mobile smartphone) and slow internet connection (Syahrizal & Pamungkas, 2021). However, these problems are not specific to FlipGrid. Lack of appropriate equipment and a slow internet connection can raise technical issues while using any web platform.

Coming to the end of this section, we must acknowledge that the literature review presented is by no means exhaustive. Other databases could also be included in the search (e.g., Web of Science), and other papers that use FlipGrid as the main research tool may have been missed due to the specific search methodology. Furthermore, it has to be mentioned that this review relies solely on scientific literature, and it may be the case that educators also use FlipGrid in other fields and in different ways than those reported in research findings.

## Using and Evaluating FlipGrid as a Tool for Practicing and Assessing Oral Science Communication. Methodology: Educational Setting, Data collection Methods & Hypotheses to be Tested

In the academic semester of 2020–2021 lectures in Greek Universities were almost exclusively carried out via video conferencing platforms (e.g., Zoom, Webex, Microsoft Teams, etc.). FlipGrid was adopted for an oral assignment in a first-year course named “Internet technologies and Design in the World Wide Web” in the Department of Communication and Digital Media at the University of Western Macedonia, Greece. The department syllabus contains a broad range of Communication lessons and lessons related to Digital Media used for communication purposes (Internet, social media, Web 2.0 tools, image & video editing, interactive multimedia technologies, etc.). It is vital that the students of this Department understand the scientific and technical information related to their field of study. It is also important that they can convey this information orally.

The assignment was about “Search Engines” and specifically the Google Search Engine and how it operates (crawling the web to track web pages, storing and indexing the information, delivering search results to the users on demand, etc.). The topic was covered in class, and students were additionally given various sources to read and a relevant online video to watch (by Google engineer Cutts, 2017). Students then had to recall what was taught in class, read, or watch the material from the given sources, understand the concept, combine the necessary information, and then organize their thoughts to create a short explanatory video of three minutes maximum on how Search Engines work. Students were also given guidelines on how to present their answers. They were instructed to try to understand the topic and present it in their own words, as clearly and concisely as possible without reading notes.

The assignment expectations were the following:

- Students should be able to communicate orally and correctly the scientific information of the concept
- The student videos would increase the educator’s awareness of their understanding of the concept
- Students could reflect on their recordings to spot their strengths and weaknesses regarding their oral performance
- The educator would also be able to pinpoint the strengths and weaknesses of each student regarding verbal communication
- The educator would get to see and hear the students. Due to the size of the class and the distance education setting adopted due to COVID-19 situation, this has been impossible during the semester. Maintaining eye contact with a large audience in video conferencing platforms such as Zoom is not feasible as the lecturer has to constantly switch between camera windows to achieve this. Not to mention that many students keep the cameras turned off (Katz & Kedem-Yemini, 2021).

A topic was created for this assignment, and the lecturer uploaded a short video explaining the assignment. The FlipGrid assignment was then embedded in Google Classroom and in a class that was created for the specific lesson—“Internet technologies and Design in the World Wide Web”. The number of students that participated in this assignment was 116. The assignment was obligatory and counted towards the final mark. The topic settings were configured to forbid students from accessing the videos of other students. So, students could not view or comment on other student videos.

Students were given 17 days to complete the assignment. At the same time, the platform was also open for student-to-teacher communication and questions regarding the assignment. It is also worth mentioning that every student received the educator’s feedback regarding their assignment performance. The educator addressed misconceptions about the topic and weaknesses in the oral presentations.

After the assignment deadline, a questionnaire was distributed to the students via Google Forms. The questionnaire consisted of Likert scale questions derived from Mohammadi’s (2015) paper. In this paper, Mohammadi attempted an integration of TAM and IS success model and relied on previous research to accomplish this (e.g., Chiu & Wang, 2008; DeLone & McLean, 2003; Hassanzadeh et al, 2012; Lee, 2010; Lin, 2007; Wang & Liao, 2008). The Information System (IS) success model was initially presented by DeLone and McLean (1992) and has been updated over the years. The updated version published in 2003 identified six components of IS success as follows: system quality, information quality, service quality, intention to use/use, user satisfaction, and net benefits model (DeLone & McLean, 2003). According to Mohammadi (2015), the revised version is one of the most widely used models for IS success, and it is the most frequently adopted to examine e-learning system success.

On the other hand, the Technology Acceptance Model (TAM) proposed by Bagozzi et al. (1992) appears to be the most widely used innovation adoption model. This model has been used in various studies to explore the factors affecting individuals’ use of new technology (Venkatesh & Davis, 2000). Davis (1989) suggests that the sequential relationship of belief–attitude–intention–behavior in TAM, enables us to predict the use of new technologies by users.

The questionnaire used for this study targeted aspects such as Ease of Use, Usefulness of the platform as a tool for developing oral skills, Satisfaction, and Intention to Reuse. Students were also asked to express freely in writing their thoughts about the platform in an open-ended question. The questionnaire was disseminated first to a small audience familiar with the FlipGrid platform (five students and two educators) to check whether the questions were understandable. All students were asked to fill out the questionnaire, including those who didn’t participate in the assignment in order to understand whether this abstention was related to the platform used. It was announced to students that a small bonus would be given for participating in the survey. The bonus counted toward the final mark.

The answers received by the online questionnaire were 137. The number of students that used FlipGrid for the assignment as mentioned was 116 and 112 of these students took part in the survey. The students that didn’t use FlipGrid and participated in the survey (25 students) were simply forwarded to a single question asking them whether their abstention had anything to do with the software (e.g., difficulties encountered). After examining their answers, it was revealed that the software had nothing to do with this abstention in all cases. The main reason expressed for not handing in the oral assignment was a lack of time.

The hypotheses that will be tested in the next section using Structural Equation Modelling are the following:

- H1. Perceived Ease of use affects Perceived Usefulness
- H2. Perceived Ease of Use affects Satisfaction
- H3. Perceived Ease of Use affects Re-usage intention
- H4. Perceived Usefulness affects Satisfaction
- H5. Perceived Usefulness affects Re-usage intention
- H6. Perceived Satisfaction affects Re-usage intention

## Results and Discussion

**Fig. 7:**
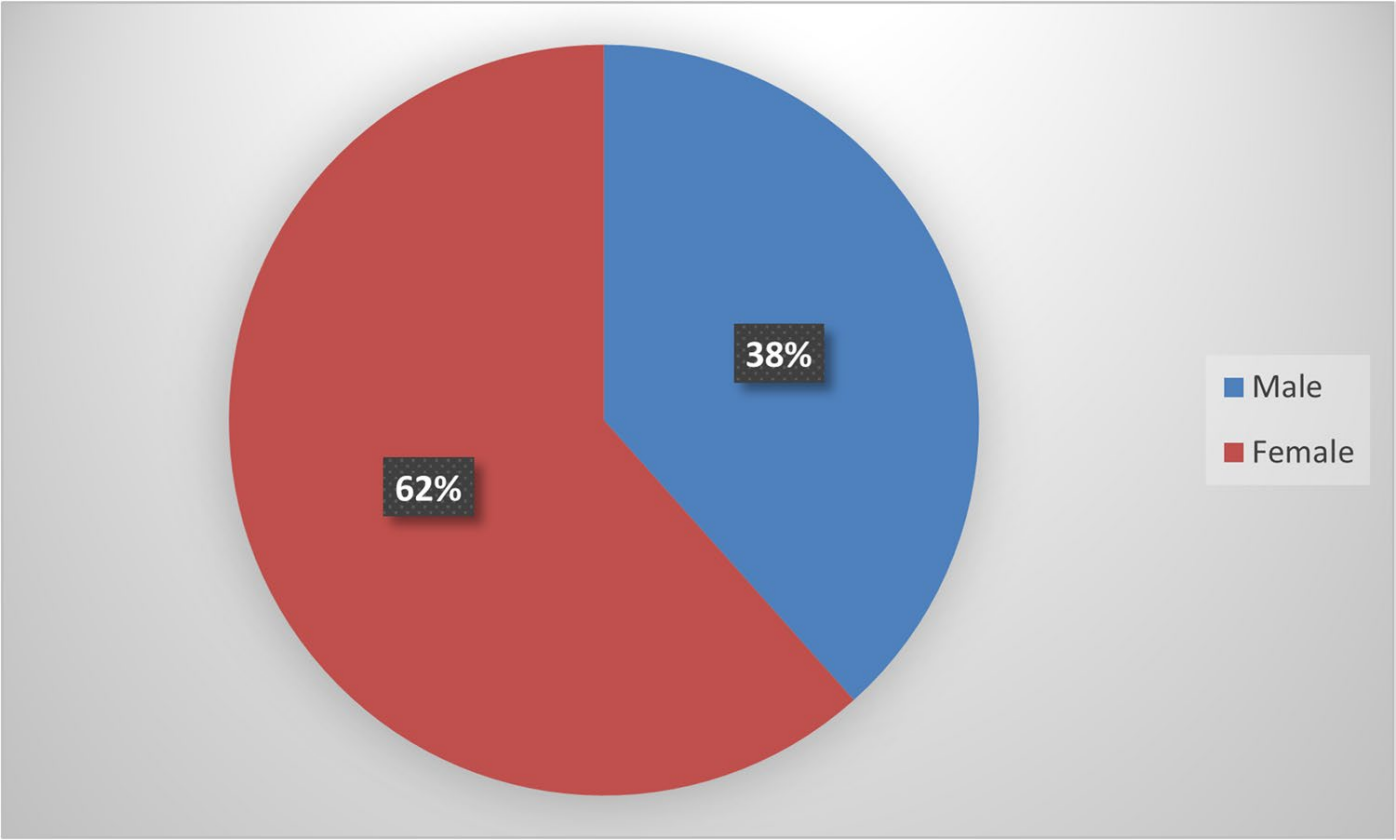
Gender

Out of the 112 first-year students that completed the assignment, 69 were female, and 43 were male, as depicted in Fig. 7.

**Fig. 8:**
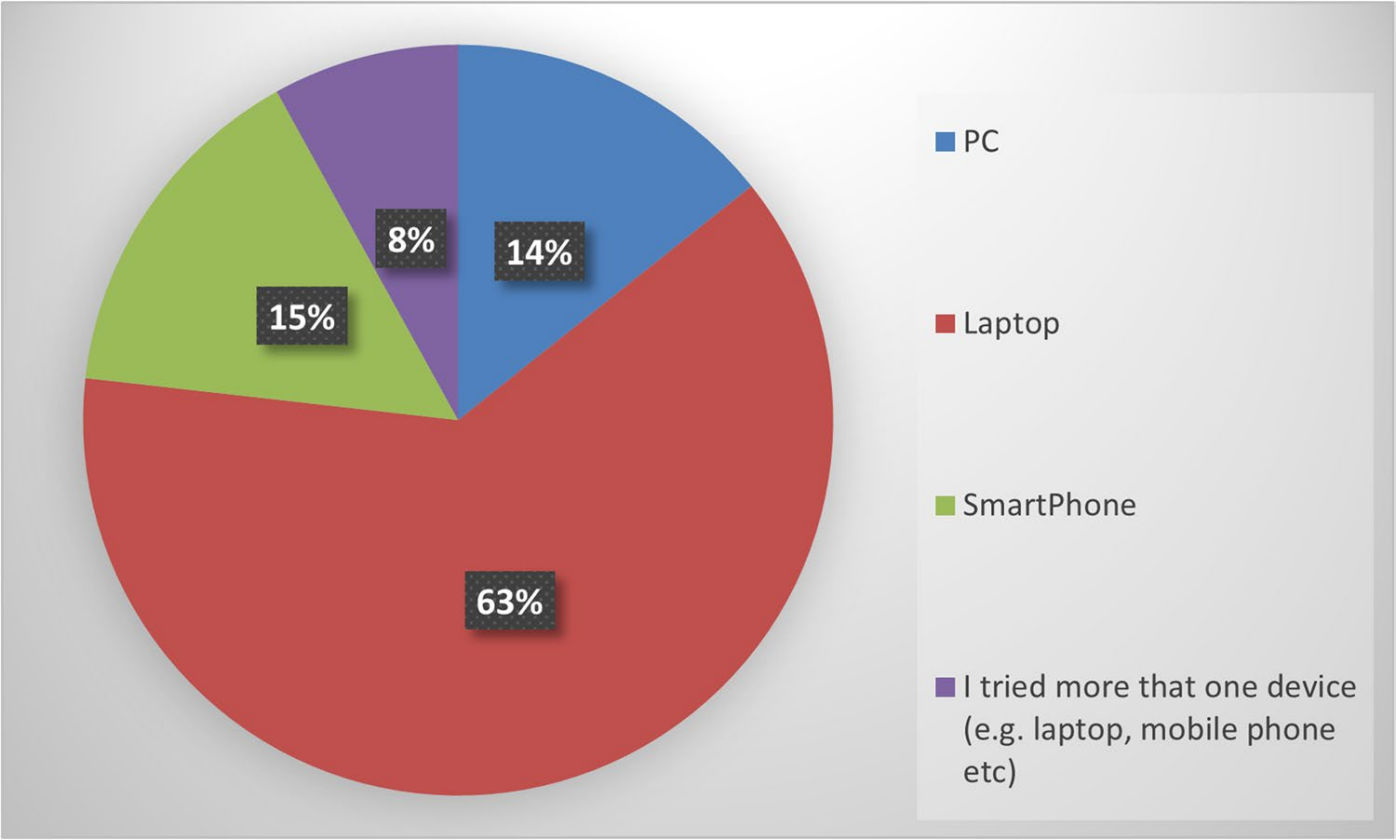
Devices Used

Regarding the age of the students, most students were between 19 and 21, apart from four mature students that were in the age range of 50 to 55. Most students used a laptop computer to complete the assignment with FlipGrid, as depicted in Fig. 8.

As mentioned, to measure perceived ease of use, perceived usefulness, satisfaction, and re-usage intention the scales used by Mohammadi (2015) were utilized and modified to fit the case of FlipGrid as a tool for oral assignments and communication with professors. The items used are presented in Table 1. All items of the four study’s scales were evaluated on five-point Likert scales ranging from (1) “strongly disagree” to (5) “strongly agree.”

**Table 1:** Descriptive Statistics, Validity and Reliability measures, and Standardized Factor Loadings

	Mean	Standard Deviation	Standardized factor loadings
Perceived Ease of use (a = 0.849; CR: 0.845, AVE: 0.53)			
FlipGrid is easy to use	4.29	0.78	0.706
FlipGrid is easy to learn	4.40	0.72	0.701
FlipGrid is easy to access	4.63	0.60	0.526
FlipGrid is easy to understand	4.45	0.67	0.814
FlipGrid is convenient	4.28	0.76	0.845
Perceived Usefulness (a = 0.848; revised scale a: 0.844, CR: 0.823, AVE: 0.49)			
FlipGrid helps to save time for the preparation of oral exercises*	4.05	0.82	
*FlipGrid helps to save time for communicating with my professor*	4.27	0.86	
FlipGrid helps me to rely on my communication skills	4.18	0.86	0.720
FlipGrid helps me to improve my communication skills	4.33	0.81	0.841
FlipGrid helps me improve my performance in oral presentations	4.30	0.76	0.687
FlipGrid is effective	4.32	0.65	0.557
Flipgrid is efficient	4.31	0.63	0.653
Satisfaction (a = 0.830, CR: 0.877, AVE: 0.65)			
Flipgrid is enjoyable	3.75	0.92	0.558
I am pleased enough with FlipGrid	4.09	0.73	0.886
FlipGrid satisfies me	4.04	0.73	0.898
FlipGrid is pleasant to me	3.89	0.90	0.754
*FlipGrip gives me self-confidence*	3.63	1.03	
Re-usage intention (a = 0.821, CR: 0.820, AVE: 0.60)			
I intend to use FlipGrid in the future for oral exercises	3.84	0.94	0.817
I intend to use FlipGrid for communication with my professor	3.93	1.01	0.793
I am likely to use FlipGrid in the near future	3.99	0.90	0.720

^*^Items in italics were dropped from further analysis

Hypothesis testing was conducted through structural equation modeling using Amos 8.0. First, the fit of the measurement model was evaluated. Then, structural equation modeling was performed to test the hypotheses.

### Statistical Analysis

The measurement model consisted of four latent variables (perceived ease of use, perceived usefulness, satisfaction, and re-usage intention) and showed acceptable fit based on the values of goodness-of-fit indices [χ2: 231.83, p = 0.000, χ2/df = 1.633; Comparative-Fit-Index (CFI) = 0.923; Incremental Fit Index (IFI) = 0.924; Tucker-Lewis-Index (TLI) = 0.907; Root Mean Square Error of Approximation (RMSEA) = 0.075]. The values of CFI, IFI, and TLI indices were above the 0.90 threshold (Byrne, 2010) and the RMSEA was lower than the 0.08 maximum value (Kline, 1998). The standardized factor loadings of all items were significant (p < 0.05) and above the 0.50 value (Janssens et al., 2008) and their critical ratios were above 1.96 except for the items “FlipGrid helps to save time for communication with my professor”, “FlipGrid helps to save time for preparation of oral exercises”, and “FlipGrip gives me self-confidence” that did not reach the 0.50 criterion and were dropped from further analysis.

After dropping the above items, the goodness-of-fit measures of the revised measurement model showed an acceptable fit (χ2: 179.91, p = 0.000, χ2/df = 1.636; CFI = 0.934; IFI = 0.936; TLI = 0.918; RMSEA = 0.076). Cronbach’s α coefficients of all factors ranged from 0.821 to 0.849, thus showing good internal reliability (> 0.70, Nunnally and Bernstein, 1994). Satisfactory composite reliabilities were observed for all scales as they ranged from 0.820 to 0.877 (> 0.70, Hair et al., 2009). Moreover, the average variance extracted (AVEs) for all latent variables was above 0.50 (Fornell & Larcker, 1981).

Table 1 shows the mean values, the standard deviations, standardized factor loadings of the items, as well as measures of reliability for the scales used in the study.

Based on the mean scores of items comprising each scale it can be argued that FlipGrid was evaluated by students as an easy-to-use and useful application for conducting oral assignments. Moreover, looking at the mean scores of satisfaction’s items, students were satisfied to a high extent by FlipGrid. These findings are similar to the many studies that revealed positive student attitudes towards the use of FlipGrid in fields such as language learning (EFL & ESL) and community building (e.g., Innes, 2020; Lowenthal & Moore, 2020; Mango, 2021; Shin & Yunus, 2021). However, moderate evaluation scores were associated with the hedonic dimension of FlipGrid and the feelings of joy and pleasure that arose through its usage. This could be attributed to the fact that FlipGrid was used as an assessment tool in this study and not as a tool for community building or entertainment.

In a similar vein, the re-usage intent of FlipGrid by students for learning and communication purposes was moderate. This also may be attributed to the fact that the tool was used for assessment purposes. It may also be the case that the assignment’s difficulty has affected the students’ views regarding the hedonic dimension and the re-usage intent. However, this is only an assumption, and the moderate re-usage intent needs further investigation. It is worth stating that these findings differ from the study of Shin and Yunus (2021). Shin and Yunus (2021) is the only study amongst the research papers examined in the literature review that uses questions from a TAM model (Davis, 1989) to explore the attitudes of primary school pupils towards using Flipgrid for learning English speaking skills. In this study, student responses regarding their attitude toward the use of FlipGrid were highly positive.

### Hypotheses Testing – Structural Model

To examine the hypothesized relationships, structural equation analysis was employed, since the goodness-of-fit measures of the measurement model were satisfactory. The structural model showed acceptable fit (× 2 = 202.33, p = 0.000, × 2/df = 1.619; CFI = 0.929, TLI = 0.913, IFI: 0.931, RMSEA = 0.075). To test the existence of common method bias, a second model was tested were all items loaded on one latent factor and their regression paths were set to be equal while the variance of the common latent factor was set to 1. Then the square of the unstandardized loading of the latent factor of each path was 0.227 [(0.477)2] and less than the 0.50 criterion (Eichhorn, 2014). Thus, common method variance was not an issue in the study’s model. Results of the hypotheses testing are shown in Table 2.

**Table 2:** Effects of Model’s Relationships

Relationship	Hypothesis	Standardized Direct Effect	Critical Ratio	p-value
Ease of use → Usefulness	H1	0.510	4.321	0.000**
Ease of use → Satisfaction	H2	0.609	4.289	0.000**
Ease of use → Re-usage intention	H3	-0.197	-1.306	0.192
Usefulness → Satisfaction	H4	0.205	1.953	0.051*
Usefulness → Re-usage intention	H5	0.602	4.564	0.000**
Satisfaction → Re-Usage Intention	H6	0.341	2.200	0.028**

^**^ < 0.05, * < 0.10

Ηypotheses H1 to H3 regarding the impact of perceived ease of use of FlipGrid on perceived usefulness, satisfaction, and re-usage intention were examined. Perceived ease of use was found to be significantly (p < 0.05) and positively related to perceived usefulness of FlipGrid (b = 0.510, p = 0.000) and the satisfaction derived by students (b = 0.609, *p* = 0.000). Thus, H1 and H2 could not be rejected. However, the impact of perceived ease of use on re-usage intention was insignificant (b = -0.197, *p* = 0.192). Hence, H3 was rejected.

Regarding H4, a marginally significant (*p* < 0.10) and positive relationship was found between perceived usefulness and students’ satisfaction (b = 0.205, *p* = 0.051) thus supporting H4. Moreover, intention of students to re-use FlipGrid was significantly (*p* < 0.05) affected in a positive way by perceived usefulness (b = 0.602, *p* = 0.000) and satisfaction (b = 0.341, *p* = 0.028). Therefore, H5 and H6 are supported.

**Fig. 9:**
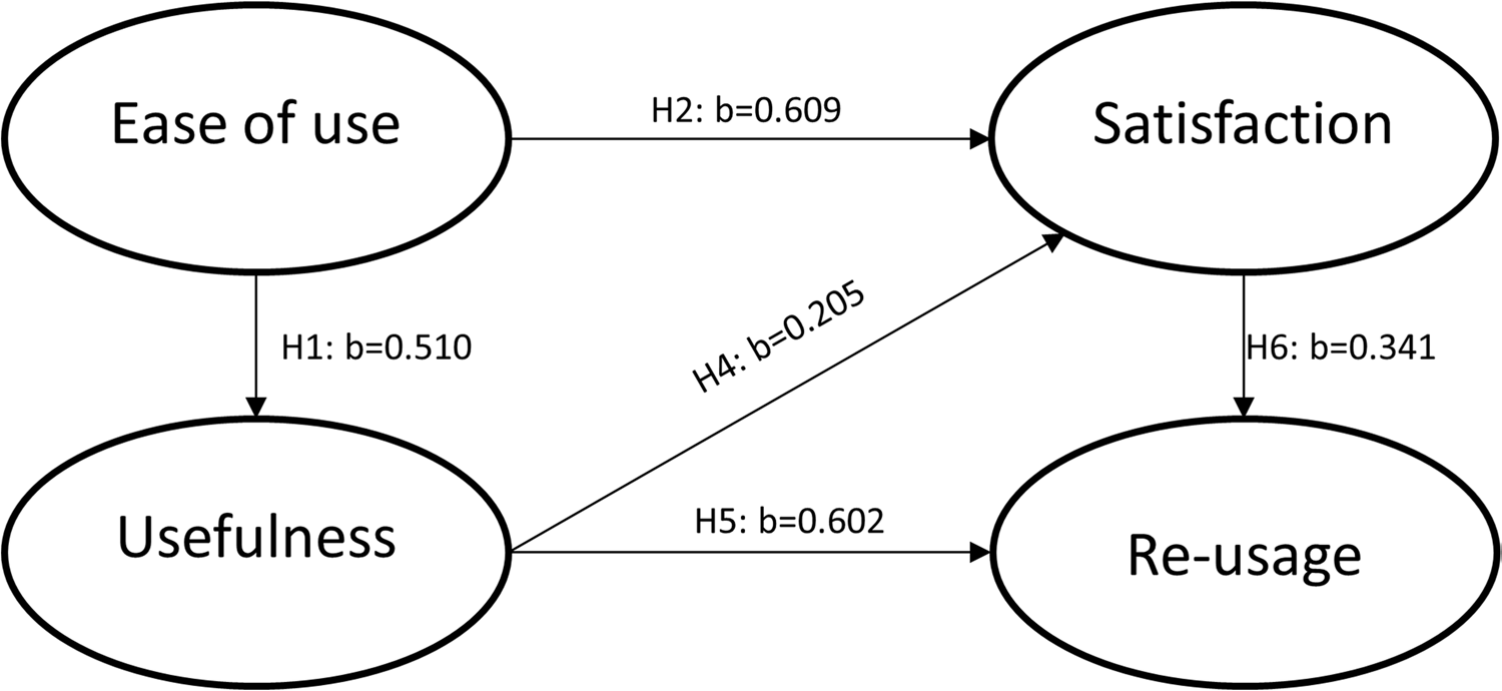
Summarizing the results of hypothesis testing

The summary of the results is depicted in Fig. 9.

These findings are similar to the results of previous studies regarding the impact of the perceived usefulness of digital technology for educational purposes on usage intention. In their study, Scherer et al., (2019) followed a meta-analytic structural equation modeling approach in TAM studies to explain teacher adoption of digital technology in education. One of the study findings was that the effects on behavioral intention were much more profound for Perceived Usefulness than for Perceived Ease of Use. Perceived Usefulness of technology seems to be a critical factor in user intentions. The authors propose that teacher education and professional development practices consider strengthening Perceived Usefulness next to Perceived Ease of Use.

### Educator Perceptions and Analysis of the Student Answers to the Open-Ended Question

Besides the oral communication practice, another benefit related to the use of FlipGrid is that the lecturer was given a chance to see his students talking and get to know them briefly, as only a percentage of them participated in the lessons by expressing their ideas and thoughts. In a large audience, such as the audience of the particular class (ranging from 110 to 150 students), it is impossible for everyone to be given the opportunity to talk or to be involved in a task that requires oral communication. The lecturer also pinpointed how well the students grasped the specific concept and the strengths and weaknesses of each student in oral communication. Students also had the chance to discover their strengths and weaknesses by reflecting on their video recordings.

As mentioned, the students were also asked to express their opinion regarding their experience with the platform in an open-ended question. The number of students that expressed their opinion in the open-ended question was 98 out of the 112 that used FlipGrid to complete the assignment. The responses were gathered and analyzed. There were two types of comments. Comments expressing solely positive opinions about the platform, and comments that expressed positive opinions but also difficulties that were encountered. The students identified some software shortcomings and made proposals about helpful features that could be incorporated into the platform. There were no responses that expressed solely negative opinions. More specifically, 57 responses contained only positive comments. The rest 41 answers contained positive comments but also comments about difficulties, shortcomings, and considerations about the software features. Furthermore, 19 responses out of the 98 contained positive comments that were related to the efficiency of FlipGrid as a tool for improving oral communication skills. Some of these comments are given below:

“FlipGrid is an easy-to-use and efficient platform for oral assignments. Especially for us, students of a communication department, it is very useful to cultivate our presentation and communication skills”

“What I liked about this assignment is that it challenged me to sit in front of a camera and to speak with confidence about a scientific subject”.

“I think that FlipGrid will help with oral assignments, familiarity with the camera, and communication in general. In my contact with the teaching staff, however, I prefer face-to-face communication.”

“I believe that FlipGrid has helped me improve my communication skills. I also think that it affected me a lot psychologically by strengthening my confidence while speaking in front of a camera. I usually don’t feel confident speaking in front of the camera, but after completing this assignment, I was able to deal with this”.

Difficulties expressed in the student comments were mainly related to technical issues. One problem reported was related to the FlipGrid mobile application. Students that used the mobile app to create their videos mentioned that the produced video did not appear anywhere in the application interface, causing confusion as to whether the video was stored or not and if it was delivered to the educator. Some students also reported slow processing and upload time. There were also students who expressed the opinion that FlipGrid has limited editing capabilities and narrows their potential to create good presentations. FlipGrid has only the capability of trimming the video from the beginning and the end, and some students reported that they would like to have the option to cut frames at any point of the video, an option that exists in most video editing software tools. It is also worth mentioning that the students did not mention anxiety issues caused by using a video recording camera tool, but we also have to keep in mind that their videos were hidden by their peers, and they were only visible to the educator.

## Summary of Findings and Limitations of the Study

Oral communication and presentation skills are essential for many academic fields and professions today, including science-related ones. Allocating sufficient time, however, for practicing these skills in the curriculum is not always possible. Large classes and time constraints are two obstacles to accomplishing this. Asynchronous video platforms can help solve this problem, and FlipGrid is a platform of increasing popularity that can be used for hosting communication tasks such as discussions, debates, oral assignments, etc. FlipGrid has become popular in the educational community due to the fact that it is free and offers some unique capabilities for both students and educators. FlipGrid is also a tool that is being continuously upgraded over recent years. It is also worth mentioning that as the writing of this research paper is in its final stages, some new features have been added to the platform, and FlipGrid is renamed to Flip (June 2022).

In the first part of this study, a detailed literature review was conducted to understand how educators use FlipGrid around the world. Although there are also other studies that review the literature regarding this tool (e.g., Green et al., 2021) to the best of our knowledge this is the only study so far that examines the literature in a systematic way. From the analysis of the publications, it became evident that the FlipGrid platform is used mainly in English language learning (ESL/EFL) and to a lesser extent in other fields such as Physical Education, Engineering, Nurse Education, Pharmacy, and Economics/Business. This research could not spot any cases of FlipGrid use in traditional scientific fields such as Maths, Physics, Chemistry, and Informatics. FlipGrid is also not used in fields that could benefit most from such a platform, such as Communication-related studies (e.g., Journalism studies). There is also a lack of studies regarding FlipGrid that utilize large samples (e.g., ≥ 100 participants). Another observation is that FlipGrid use increased in the years of the COVID-19 pandemic.

There are, however, also limitations regarding this literature review. As already mentioned, the search methodology isn’t exhaustive. Other databases could also be included in the search (e.g. Web of Science) and articles found could be searched for references to other relevant articles. Furthermore, it has to be said that only the scientific literature is explored. There is a possibility that FlipGrid is used more extensively by educators who do not conduct research and thus do not publish their experiences and findings in journals and conference proceedings. As future research other sources could also be explored to obtain a broader picture of how FlipGrid is used, such as educational blogs and related social media groups. FligGrid, as mentioned in the introduction, has an active community on Twitter, and there are thousands of posts with the hashtag #FlipGrid.

In the second part of this study, the efficiency of FlipGrid as a tool for practicing oral science communication was also evaluated. More specifically, FlipGrid was used to assess students’ ability to convey scientific information in an informatics-related subject. To the best of our knowledge, it is the first study that the TAM model is applied to test the interrelationships between perceived ease of use, perceived usefulness, satisfaction, and re-usage intention of FlipGrid using structural equation modeling. Results showed that students evaluated FlipGrid as an easy-to-use and useful application for improving communication skills, and they were satisfied to a high extent by FlipGrid. However, moderate evaluation scores were associated with the hedonic dimension of FlipGrid and the feelings of joy and pleasure that arose through its usage. This finding could be attributed to the fact that in this study FlipGrid was used as an assessment tool and not as a tool for community building, entertainment, or less demanding activities such as a video forum for posting and answering questions about a lesson.

Similarly, the re-usage intent of FlipGrid for learning and communication purposes was moderate. This may again be attributed to the fact that the tool was used for assessment purposes. It may also be the case that the assignment’s difficulty has affected the students’ views regarding the hedonic dimension and the re-usage intent, but this should be further investigated.

Several hypotheses were tested using structural equation analysis, and perhaps the most important observations are the following: a) student satisfaction and the perceived usefulness of the tool were found to be significantly and positively related to the perceived ease of use of FlipGrid and b) intention of students to re-use FlipGrid was significantly affected positively by perceived usefulness and satisfaction. The study’s findings corroborate the results of previous studies regarding the impact of the perceived usefulness of digital technology for educational purposes on usage intention (Scherer et al., 2019).

Students were also asked to comment freely on the platform’s efficiency, and the comments received consisted mainly of positive impressions regarding the platform’s usefulness. However, there were also comments regarding technical difficulties encountered as well as proposals of how the software could be improved.

Finally, another limitation of the study concerns the sample used. Although the study is amongst the few studies that utilize a sample of more than 100 students, the sample size is still relatively small and consists of students coming from a certain geographical area. Thus, there is a need for more studies that involve large samples and students from different countries.

## Conclusion

FlipGrid is a popular platform that is mainly used for improving oral skills (mainly in EFL/ESL) through self-reflection and feedback from the instructor or peer students and for initiating discussions that contribute to community building. Although the use of FlipGrid as a tool for developing oral science communication skills is not widespread and, in many fields, it is nonexistent (e.g., informatics, physics, math, etc.), in this study, FlipFrid proved to be an easy-to-use platform, a platform that satisfied the students, as well as a useful platform for practicing oral science communication.

## Appendix A

**Table 3:** Publications

	Author and Year	Field	Aim of research	Research methods & data collection instruments	Educational Level	Country	Sample size
1	Tuyet and Khang (2020)	EFL, ESL	The paper aims at investigating whether Flipgrid reduces speaking anxiety in EFL high school learners. The study also explores the learners’ attitudes towards the use of FlipGrid	Quasi-experimental method and mixed methods approach. The data collection instruments were the following: modified Foreign Language Classroom Anxiety Scale (FLCAS), questionnaire, and semistructured interviews	K12 (Highschool)	Vietnam	60
2	Shin and Yunus (2021)	EFL, ESL	This study aimed to investigate the attitudes of primary school students towards the use of Flipgrid in improving English speaking skills	A mixed-methods approach was adopted. Data collection was achieved with the use of a questionnaire and semi-structured interviews	K12 (Primary)	Malaysia	60
3	Budiarta and Santosa (2020)	EFL, ESL	This research aimed to explore students’ perceptions of the use of FlipGrid in an EFL speaking class as well as the benefits of implementing a combination of TPS (Think Pair Share) and Flipgrid	Qualitative research with case study design. The instruments used were an online questionnaire consisting of open-ended questions, semi-structured interview, and observation	Teacher training	Indonesia	22
4	Innes (2020)	EFL, ESL	The aim of this study was the evaluation of the FlipGrid platform by students of a Japanese University to explore what students liked and what they disliked about the tool	A qualitative survey was used consisting of open-ended questions	Higher Education	Japan	100
5	Kiles et al. (2020)	Pharmacy	This research aimed to evaluate FlipGrid as a self-reflection tool	Quantitative and qualitative analysis. Online questionnaires consisting of open-ended questions and questionnaires consisting of Likert-scale items	Higher Education	USA	84
6	Syahrizal and Pamungkas (2021)	EFL, ESL	This study aimed to examine student perceptions on the use of FlipGrid as a tool for improving English speaking	Qualitative method. The instruments used in this study were a questionnaire consisting of open-ended questions and interviews	K12	Indonesia	177
7	Mango (2021)	EFL, ESL	This study aimed to investigate students’ perceptions about the advantages and disadvantages of FlipGrid in learning Arabic as a foreign language	Quantitative and qualitative analysis. Data collection was achieved through a survey composed of Likert-scale items and open-ended questions	Higher Education	USA	30
8	McLain (2018)	EFL, ESL	This paper discusses the need and rationale for using FlipGrid in business English writing classes The study also evaluates the platform	The evaluation model used was the Transactional Model. Boulmetis and Dutwin (2014). The Data sources were: Instructor Observations, Survey, FlipGrid Data (e.g. responses, length of videos, etc.)	Higher Education	Korea	133
9	Taylor and Hinchman (2020)	Physical Education	This paper explores how Flipgrid could enhance classroom instruction and students’ understanding of diverse topics of kinesiology	Presentation of Strategies	Higher Education	USA	
10	Wilson et al. (2021)	Pharmacy	This paper investigates student perceptions of the use of FlipGrid in an activity to share a simulated patient experience in a non-prescription aisle	Qualitative analysis. Students completed a questionnaire regarding their perceptions about FlipGrid	Higher Education	USA	68
11	Damayanti and Citraningrum (2021)	EFL, ESL	The study aimed to explore the use of Flipgrid’s as a tool for improving speaking skills	Qualitative data analysis. The data were obtained from students’ speaking videos and interviews	Higher Education	Indonesia	19
12	Stoszkowski and Collins (2021)	Sports Coaching (Physical Education)	This study aimed to develop preliminary theories regarding the settings under which the Flipgrid facilitatesand promotes more analytical interaction between students when compared to online written blogs	Mixed-metho approach (qualitative & quantitative). Data was collected through surveys, a semi-structured focus group interview, participant observation, and content analysis of video transcripts	Higher Education	UK	21
13	Bauler (2021)	EFL, ESL	This study analyzes freely available teaching materials that were designed specifically for the Flipgrid platform	Teaching materials were selected from public and available sources on the web. A critical discourse analysis followed	Higher Education & K12	USA	-
14	Serembus and Murphy (2020)	Nurse Education	This study aimed to explore the use of Flipgrid as a platform for promoting collaboration and student engagement in a graduate nursing course	A qualitative survey comparing online text-based discussions to video discussions	Higher Education	USA	23
15	Ismail and Omar (2020)	Engineering	This study aimed to evaluate the Flipgrid as a platform for hosting short video assignments	Feedback process was carried out by using questionnaires to gauge students’ satisfaction and their experience	Higher Education	Malaysia	83
16	Edwards and Lane (2021)	EFL, ESL	This paper explores the student perceptions about the Flipgrid platform as a tool for facilitating interaction in an English communication course	Quantitative and qualitative data was gathered using online surveys consisting of Likert-scale items and open-ended questions. Results were derived from a statistical analysis of the Likert-scale items and a content analysis of the open-ended survey questions	Higher Education	Japan	189
17	Holbeck et al. (2018)	Educator Perceptions, Teacher Education	This paper explores the experiences of a full-time faculty member on the use of Flipgrid	Presentation of teacher perceptions	Higher Education	USA	
18	Carr and Krugge (2020)	Economics	This study aimed to explore teacher’s perceptions on the use of the Flipgrid platform in teaching economics education	Qualitative methods approach. Interviews were used to explore teacher perceptions	K12-Primary	USA	1
19	Petersen et al. (2020)	EFL, ESL	The purpose of this study is to evaluate the efficacy of the use of ‘Flipgrid,’ in practicing English communication	Qualitative and quantitative analysis. The instruments for data collection were a questionnaire for recording student responses and teacher observations. Video data were also used	Higher Education	Japan	37
20	Lowenthal and Moore (2020)	Educational Technology	The purpose of thisthe study was to investigate students’ perceptions of the use of Flipgrid as a tool for conducting asynchronous video-based discussions in online courses	Qualitative and quantitative analysis. Data was collected using a survey consisting of Likert-scale and open-ended questions	Higher Education	USA	79
21	Green et al. (2021)	Educator Perceptions	This study aimed to investigate how educators are using Flipgrid and their perceptions about the tool	An exploratory study using simple descriptive statistics. Data gathering was done via an online survey consisting of Likert scale items, and close-ended & open-ended questions	Teacher training & Teacher Perceptions	USA	230
22	Molina and Guillen (2021)	Educator Perceptions, Teacher Education	The study examines the beliefs of prospective teachers concerning the usability of educationaltechnologies (EdTech) and specifically the Flipgrid platform and Padlet	Quantitative data was collected using a Likert scale questionnaire and semi-structured digital-base interviews were carried out as a qualitative research method	Teacher training & Teacher Perceptions	Spain	96
23	Iglesias (2021)	EFL, ESL	This paper aimed to record the student experiences from a collaborative project using FlipGrid and Blogger	A methodological mixed-methods approach was adopted. More specifically a bibliographic exploration related to FlipGrid and Blogs was conducted. Data that was collected from FlipGrid and Blogger was examined and a questionnaire was used to record learner satisfaction	Higher Education	Spain	17
24	Keiper et al. (2021)	Economics/Business	The purpose of this paper was to investigate the perceived effectiveness of Flipgrid, in HyFlex delivery modality business courses	Quantitative and qualitative analysis. A questionnaire containing both Likert-scale and open-ended questions was distributed to undergraduate and graduate students of business courses	Higher Education	USA	163
25	Miskam and Saidalvi (2019)	Engineering	The paper explores the effectiveness Flipgrid, as a platform, for teaching oral presentation skills to engineering students	The study utilized four instruments: 1) recorded videos, 2) questionnaire, 3) semi-structured focus group interview,4) oral presentation rubric. The data was analyzed using statistical and content analysis	Higher Education	Malaysia	25
26	Davis and Colella (2021)	Nurse Education	This paper aimed to record the experiences of nurse practitioner students’ from an assignment using FlipGrid and the Snapps Technique	Anecdotal feedback was gathered and analyzed from students and faculty members following the implementation of an assignment in which FlipGrid was used	Higher Education	USA	
27	Wood (2021)	Film studies	The purpose of this study is to record educator experience from using FlipGrid in Flim Studies classes	Observation and presentation of the experiences	Higher Education	USA	
28	Stoszkowski (2018)	Sports coaching (Physical Education)	This paper reviews the use of Flipgrid to develop social learning in an undergraduate sports coaching course	Observation and presentation of the experience and perceived usefulness of the tool	Higher Education	UK	
29	Stoszkowski et al. (2021)	Coach development (Physical Education)	The purpose of this research was to explore the potential effectiveness of Flipgrid as a tool that facilitates and promotes collaborative online learning and as a tool used for critical reflection in coach development	Video metrics (length of video, replies, number of views) were examined to record participation and engagement, and content analysis of all video transcripts was conducted in order to examine the reflective quality of the comments posted. Transcripts were coded using a specified framework	Higher Education	UK	21
